# Antiplatelet Agents Affecting GPCR Signaling Implicated in Tumor Metastasis

**DOI:** 10.3390/cells11040725

**Published:** 2022-02-18

**Authors:** Gianenrico Rovati, Annalisa Contursi, Annalisa Bruno, Stefania Tacconelli, Patrizia Ballerini, Paola Patrignani

**Affiliations:** 1 Department of Pharmaceutical Sciences, University of Milan, 20122 Milan, Italy; genrico.rovati@unimi.it; 2 Laboratory of Systems Pharmacology and Translational Therapies, Center for Advanced Studies and Technology (CAST), School of Medicine, “G. d’Annunzio” University, 66100 Chieti, Italy; annalisa.contursi@unich.it (A.C.); a.bruno@unich.it (A.B.); stefania.tacconelli@unich.it (S.T.); patrizia.ballerini@unich.it (P.B.); 3 Department of Neuroscience, Imaging and Clinical Science, School of Medicine, “G. d’Annunzio” University, 66100 Chieti, Italy; 4 Department of Innovative Technologies in Medicine and Dentistry, “G. d’Annunzio” University, 66100 Chieti, Italy

**Keywords:** cancer metastasis, G protein-coupled receptors, epithelial–mesenchymal transition, platelets, cyclooxygenases, P2Y12 antagonists, PGE_2_ receptor antagonists, TXA_2_ receptor antagonists, aspirin, antithrombotic agents

## Abstract

Metastasis requires that cancer cells survive in the circulation, colonize distant organs, and grow. Despite platelets being central contributors to hemostasis, leukocyte trafficking during inflammation, and vessel stability maintenance, there is significant evidence to support their essential role in supporting metastasis through different mechanisms. In addition to their direct interaction with cancer cells, thus forming heteroaggregates such as leukocytes, platelets release molecules that are necessary to promote a disseminating phenotype in cancer cells via the induction of an epithelial–mesenchymal-like transition. Therefore, agents that affect platelet activation can potentially restrain these prometastatic mechanisms. Although the primary adhesion of platelets to cancer cells is mainly independent of G protein-mediated signaling, soluble mediators released from platelets, such as ADP, thromboxane (TX) A_2_, and prostaglandin (PG) E_2_, act through G protein-coupled receptors (GPCRs) to cause the activation of more additional platelets and drive metastatic signaling pathways in cancer cells. In this review, we examine the contribution of the GPCRs of platelets and cancer cells in the development of cancer metastasis. Finally, the possible use of agents affecting GPCR signaling pathways as antimetastatic agents is discussed.

## 1. Introduction

Thrombosis is commonly detected in cancer patients and is associated with the progression to a metastatic stage. Signs of aberrant platelet activation, aggregation, and enhanced platelet turnover can be detected in this scenario [1,2]. Considerable evidence from experimental models has been accumulated on the functional relationship between platelets and tumor progression. Platelets can protect tumor cells from immune elimination within the circulatory system, promote tumor cell arrest within the vasculature, and affect tumor cell survival, thereby supporting the spreading of the tumor to distant organs [3]. Thus, both the pharmacological inhibition of platelet function and platelet crosstalk with cancer cells have the potential to prevent cancer metastasis development [4,5,6,7].

Heptahelical transmembrane receptors (7TMs), more commonly known as G protein-coupled receptors (GPCRs), represent the most prominent family of membrane receptors and the most versatile type of transmembrane signaling protein. They act mainly through coupling with heterotrimeric G proteins, composed of the subunits α, β, and γ, and are encoded by more than 800 genes in the human genome and divided into five main classes [8].

GPCRs, known to regulate diverse physiological processes, are implicated in some of the most frequent human diseases. GPCR/G protein-mediated signaling impacts oncogenesis at multiple levels by regulating tumor angiogenesis, immune evasion, and metastasis [9,10]. PI 3-kinase (PI3K) signaling is one of the major pathways activated downstream of GPCRs via Gbg subunits which directly bind to the p110β and p110γ catalytic subunits of PI3Kb and PI3Kg. The PI3K pathway is frequently altered in cancer [11]. Khalil et al. [12] clarified the role of Gbg–p110b interaction in the tumor cell–macrophage crosstalk and invadopodia function involved in the metastatic process. They suggested that the disruption of p110β–Gβγ binding could constitute a novel therapeutic pharmacological approach to treating metastasis [12].

Human platelets express ten members of the G_s_, G_i_, G_q_, and G_12_ families (at least one G_s_, four G_i_ (G_i1_, G_i2_, G_i3_, and G_z_), three G_q_ (G_q_, G_11_, and G_16_), and two G_12_ family members (G_12_ and G_13_)) [13]. The primary platelet stimuli, i.e., thrombin, ADP, and thromboxane (TX) A_2_, as well as the main platelet, act through the activation of specific GPCRs. Antiplatelet agents, which are direct inhibitors of GPCRs, such as ADP receptor P2Y12 antagonists, are clinically employed to reduce the occurrence of cardiovascular events in high-risk patients. However, they are associated with an enhanced risk of major bleeding, especially when given in dual antiplatelet therapy with low-dose aspirin (an inhibitor of TXA_2_ biosynthesis) to improve efficacy in patients with acute coronary syndromes and for those undergoing percutaneous coronary interventions [14]. Thus, novel agents with a safer profile are desirable. It has been suggested that targeting the intracellular signaling molecules of GPCRs might provide a novel strategy for antithrombotic therapy and possibly for the prevention of cancer and metastasis. However, the widespread presence in various cells of these signaling molecules might preclude a selective action using this pharmacological approach; this concern has limited research in this area, with those involved preferring to develop direct GPCR inhibitors. PI3K and G_q_ inhibitors are among the novel strategies under investigation to prevent the occurrence of arterial ischemic events [15].

In this review, we examine the contribution of the GPCRs of platelets and cancer cells in the development of cancer metastasis. Finally, the possible use of agents affecting GPCR signaling pathways as antimetastatic agents is discussed. 

## 2. Major G Protein-Mediated Signaling during Platelet Activation

**Figure 1:**
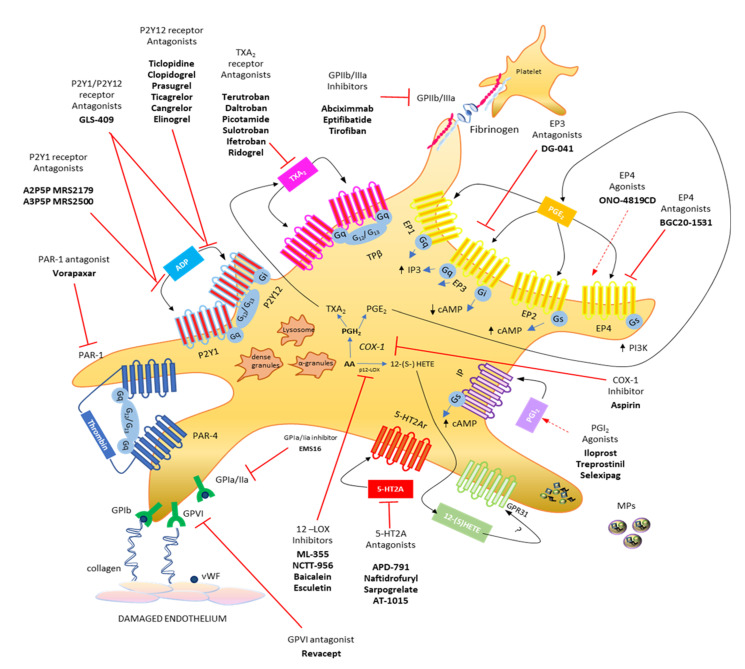
Platelet biology: main eicosanoids produced, receptors, and hypothetical therapeutic strategies. Platelets release various soluble mediators, cell adhesion proteins and growth factors stored in granules in the cytoplasm. Moreover, platelets release vesicles rich in genetic material (including microRNAs). Platelet membrane has different transmembrane receptors involved in crosstalk with other platelets and different cell types. Following endothelial damage, platelets can bind to the damaged tissue by binding integrin receptors (GP, glycoproteins) to extracellular matrix proteins, such as collagen and VWF (von Willebrand factor). Platelet activation due to the action of ADP can be influenced by using P2Y12 receptor antagonists and can be prevented by using aspirin, which irreversibly inhibits the activity of the cyclooxygenase (COX)-1 involved in the production of prostaglandin (PG)H_2_, which is then converted into the potent stimulus for platelet aggregation, thromboxane (TX)A_2_, by the activity of TX synthase (TXAS), or into prostaglandin (PG) E_2_, which is produced by the activity of several PGE synthases (PGES). Both prostanoids perform their activities by interacting with their specific receptors, TP and EP1-4, respectively. From arachidonic acid, platelets, through the action of platelet-type lipoxygenase (p12-LOX), can produce another abundant eicosanoid, 12(S)-HETE [12(S)-hydroxyeicosatetraenoic acid], which exerts its action, at least in part, by activating the orphan receptor GPR31. The activation of platelets by thrombin involves the two PAR receptors, PAR-1 and PAR-4, for which different antagonists have been reported. The most recently developed antiplatelet agents include serotonin receptor antagonists (5-HT2A antagonists). Modified from Figure 1, ref [4].

The primary response of platelets to tissue damage (such as in the vascular endothelium) is adhesion, which is a multistep process involving the interaction of platelets with the subendothelial extracellular matrix, that contains adhesive macromolecules including von Willebrand factor (vWF) and collagen, via the platelet vWF receptor GPIb/V/IX and the collagen receptor GPVI [16,17,18] (Figure 1). This response is mainly independent of G protein-mediated signaling. GPVI acts via FcR chain activation to promote the inside-out activation of integrins such as a_IIb_b_3_ (GPIIb/IIIa) or a_2_b_1_ (GPIa/IIa) [18,19], thus leading to the formation of a stable platelet monolayer. Recently, the collagen receptor GPR56/ADGRG1 (an adhesion G protein-coupled receptor) has been identified, which is a GPCR coupled to G_13_ and activated by autoproteolysis [20]. 

**Figure 2:**
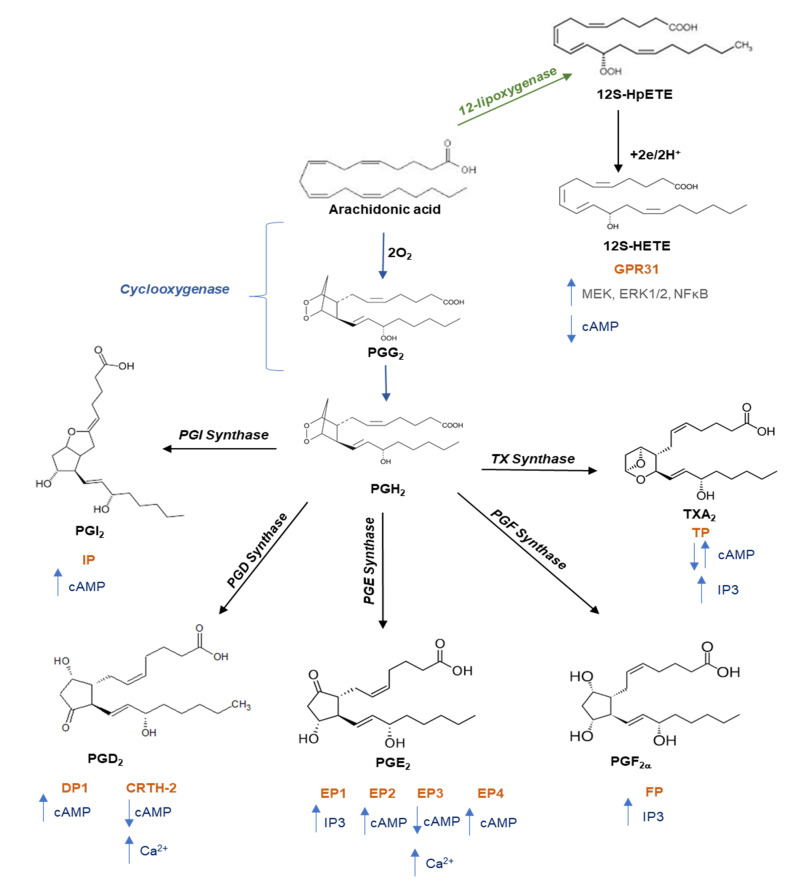
Pathway of prostanoid and 12-hydroxyeicosatetraenoic acid biosynthesis. Free arachidonic acid is metabolized into: (i) prostaglandin (PG)G_2_ and then into PGH_2_ through the catalytic activity of cyclooxygenase; PGH_2_ is then converted into prostanoids (prostaciclyn (PGI_2_), PGE_2_, PGF_2a_, PGD_2_, and TXA_2_) by tissue- and cell-specific isomerases; (ii) 12S-hydroperoxyeicosatetraenoic acid (12S-HpETE) by the activity of 12-lipoxygenase (12-LOX) and then subsequently reduced to 12S-hydroxyeicosatetraenoic acid (12S-HETE). Once formed, prostanoids and 12S-HETE bind to their specific G protein-coupled receptors: the E prostanoid receptor (EP) 1, EP2, EP3, and EP4 subtypes of the PGE_2_ receptor; the PGD receptor (DP1 and CRTH-2); the PGF_2a_ receptor (FP); the PGI_2_ receptor (IP); the thromboxane receptor (TPa and TPb), and GPR31. These receptors activate various intracellular signaling pathways which mediate the effects of receptor activation on cell function. Abbreviations: CRTH-2, chemoattractant receptor-homologous molecule expressed on T helper 2 cells; MEK, mitogen-activated protein kinase kinase; ERK, extracellular-signal regulated kinases; NFkB, nuclear factor kappaB; cAMP, cyclic adenosine 3′,5′-monophosphate; PI3K, phosphatidylinositol 3-kinase; IP3, inositol trisphosphate.

Subsequently, additional platelets are recruited, forming a growing platelet thrombus. To this response, the contribution of some soluble mediators released from platelets, such as ADP (stored into the dense granules), TXA_2_ and prostaglandin (PG)E_2_ (generated from arachidonic acid (AA) via the activity of cyclooxygenase (COX)-1), and 12S-hydroperoxy-eicosatetraenoic acid (12S-HETE) (produced from AA by the 12S-lipoxygenase (LOX)) are requested [21,22,23] (Figure 1 and Figure 2). 

ADP and TXA_2_ are the main mediators involved in the second phase of platelet activation during thrombosis and hemostasis via the activation of G protein-mediated signaling pathways [21,22]. ADP activates platelets via its interaction with two G protein-coupled receptors, P2Y1 and P2Y12 [21]. P2Y1 couples to G_q_ while P2Y12 is coupled to Gi-type G proteins [21]. The activation of both receptors is required for a full response of platelets to ADP [24]. TXA_2_ also functions to amplify the platelet activation mediated by primary agonists (such as collagen and thrombin). The TXA_2_ receptor (TP) couples to G_q_ and G_12_/G_13_ [25,26]. Platelets from TP deficient mice prolonged bleeding times and were not able to form stable thrombi [27]. 

PGE_2_ binds and activates four GPCRs, EP1, EP2, EP3, and EP4. Each of these receptors has a distinct pharmacological signature and intracellular signal transduction [28]. Stimulation of the EP3 receptors results in free intracellular Ca^2+^ levels elevation. In contrast, stimulation of the EP2 and EP4 receptors usually increases intracellular cAMP levels through the activation of the Gs protein, resulting in a decrease in intracellular Ca^2+^ levels. The EP3 receptor expressed on platelets is coupled to G_i_-type G proteins [29]. In mice lacking EP3, bleeding times were increased, and the potentiating effects of PGE_2_ were abrogated [30].

Thrombin has an important role as an effector protease of the coagulation system and is among the most effective activators of platelets. It acts via protease-activated receptors (PARs) [31,32], which couple to G_q_ and G_12_/G_13_.

Several other stimuli (such as serotonin and epinephrine) that act through GPCRs have been identified; these are weak platelet activators but can potentiate platelet responses to different stimuli [33].

Some mediators are inhibitors of platelets acting through GPCRs. Prostacyclin (PGI_2_), generated from AA mainly via vascular COX-2 (Figure 2), operates through a G_s_-coupled receptor, the IP receptor, which stimulates adenylyl cyclase (AC). PGI_2_-dependent platelet inhibition plays an important role in the protective cardiovascular effects of prostacyclin. PGI_2_ constrains the action of several mediators that activate the platelet, including TXA_2_. Thus, the selective inhibition of vascular COX-2-dependent PGI_2_ by coxibs is associated with adverse cardiovascular effects [34]. 

Another abundant eicosanoid produced by platelets is 12S-HETE, which is produced via the activity of the platelet-type 12-LOX (Figure 1 and Figure 2) [23]. The mechanism of action of 12S-HETE is not yet entirely understood. 12-HETE can stimulate NOX (NADPH oxidase) and enhance ROS production (reactive oxygen species). Recently, it has been proposed that 12S-HETE acts through the activation of the orphan receptor GPR31 [35]. Van Doren et al. [36] showed that in platelets, GPR31 is coupled to G_i_ and that its activation by 12S-HETE potentiates platelet aggregation, calcium flux, and dense granule release induced by the stimulation of PAR4 (protease-activated receptor 4) by thrombin [36]. Interestingly, they developed a GPR31 pepducin antagonist (GPR310), a synthetic 21mer peptide conjugated to palmitate to form an N-palmitoylated lipopeptide targeting the i3 loop of GPR31. GPR310 significantly reduced arterial thrombosis without affecting hemostasis in mice [36]. 

An oral selective inhibitor of platelet-type 12-LOX, ML355, has been developed and impairs thrombus formation and vessel occlusion in FeCl3-induced mesenteric and laser-induced cremaster arteriole thrombosis models with minimal effects on hemostasis [37]. Recently, He et al. (2020) developed a drug-delivery system for ML355 using a reconstituted nanoparticle, synthetic high-density lipoprotein (sHDL), which mimics the native HDL. ML355-sHDL exhibited more a potent inhibition of thrombus formation in both small arterioles and larger arteries in mice without impairing the normal hemostasis in vivo [38]. 

## 3. Role of Platelets in Tumorigenesis and Metastasis

The dissemination of primary epithelial tumor cells to distant organs occurs via the bloodstream and lymphatics. It depends on acquiring a disseminating phenotype that allows tumor cells to colonize distant organs, and the interaction of tumor cells with the host microenvironment plays a critical role in this [3,39]. Within the bloodstream, tumor cells form heteroaggregates with platelets that also include leukocytes [40] which support tumor cell survival and protection from immune elimination [41]. 

The role of platelets in supporting metastasis has already been illustrated [42,43,44]. In addition to their contribution to tumor cell arrest, survival, and immune evasion [3], platelet–tumor cell interaction reprograms tumor cells to a more invasive, mesenchymal-like, and aggressive phenotype in experimental lung metastasis models [42,43,44].

**Figure 3:**
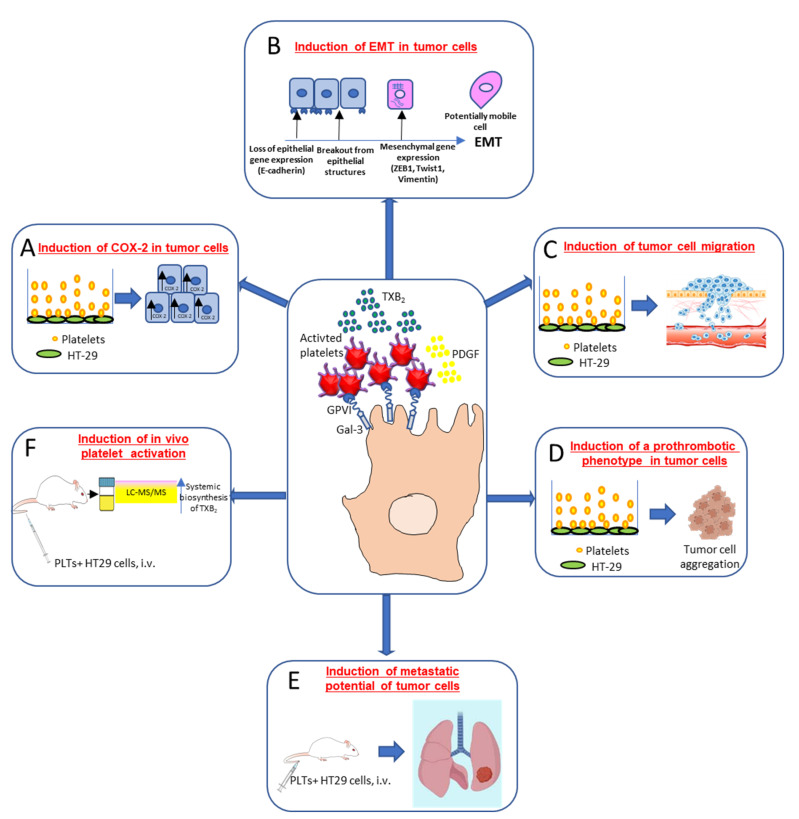
Interaction of cancer cells with platelets: molecular and functional consequences. Platelet/cancer cell crosstalk involves the interaction between collagen receptors, mainly glycoprotein (GP)VI (expressed on platelets) and galectin (Gal)-3, a protein highly expressed in different types of tumor cells. This interaction leads to platelet-dependent thromboxane (TX)A_2_ generation and increased COX-2 mRNA levels in colon adenocarcinoma HT29 cells (**A**). Activated platelets release other soluble mediators, such as platelet-derived growth factor (PDGF), which promotes COX-2 protein induction, a key event in the induction of epithelial–mesenchymal transition (EMT) (**B**). This process is characterized by the loss of their orientation and cell to cell contacts and the adoption of the mobile characteristics of mesenchymal cells, as well as the increased expression of EMT marker genes, such as the EMT-inducing transcription factors ZEB1 and TWIST1 and the mesenchymal marker vimentin, in association with a reduced expression of the epithelial marker E-cadherin. Platelet-induced EMT also promotes tumor cell migration in vitro (**C**). Moreover, cancer cells cultured in the presence of platelets induced complete platelet aggregation (**D**), thus strongly supporting the hypothesis that cancer cells undergoing EMT are characterized by an enhanced ability to activate platelets. An experimental model of hematogenous metastases, where immunodeficient NOD-*scid IL2Rγ^null^* (NSG) mice were injected via the tail vein with colon adenocarcinoma HT29 cells showed that tumor cell exposure to platelets in vitro caused a significant increase in the development of metastases (**E**); it was associated with enhanced platelet activation *in vivo*, as assessed by the urinary levels of TX-M, which is a major enzymatic metabolite of TXA_2_, a potent stimulus for platelet activation, mainly derived from platelets (**F**). This finding supports the idea that platelets may prime cancer cells to enhance their pro-thrombotic properties [43,44].

Dovizio et al. [43] (Figure 3) have shown that in coculture experiments, unstimulated platelets interact rapidly with the human colon adenocarcinoma cell line (HT29) through the binding of platelet collagen receptors (in particular, GPVI) and tumor components, such as galectin-3. This early event translated into platelet activation, as demonstrated by the enhanced generation of TXA_2_. Direct platelet–tumor cell interaction was associated with enhanced mRNA expression of COX-2 (but not COX-2 protein) (Figure 3A), epithelial–mesenchymal transition (EMT)-inducing transcription factors, such as ZEB1 and TWIST1, and the mesenchymal marker vimentin (VIM) (Figure 3B). Later, platelet aggregates detached from tumor cells, possibly due to the shedding of platelet GPVI receptors, and acquired the capacity to release their a-granule products, such as platelet-derived growth factor (PDGF). PDGF release was associated with COX-2 mRNA stabilization via NHE-PI3K/PKCd-dependent nucleocytoplasmic translocation of the mRNA-stabilizing protein HuR and COX-2 protein synthesis. In HT29 cells, overexpressed COX-2 and the enhanced generation of PGE_2_ emanated mitogenic and survival signaling pathways through the downregulation of p21^WAF1/CIP1^ and the upregulation of cyclin B1 as well as of EMT inducing transcription factors and mesenchymal markers, such as VIM, in association with the repression of epithelial markers, such as CDH1 (E-cadherin) [43] (Figure 3B). 

Guillem-Llobat et al. [44] have shown that the exposure of colon cancer cells with human platelets leads to the induction of mesenchymal-like cancer cells with enhanced capacity of cell mobility (Figure 3C) and a proaggregatory action (Figure 3D) on platelets in vitro and in vivo. Human HT29 cells pre-exposed to platelets in vitro and injected via the tail vein of immunodeficient NOD-scid IL2Rγnull (NSG) mice showed an enhanced ability to form lung metastases *versus* HT29 cells non exposed to platelets [44] (Figure 3E). Cancer cells exposed to platelets in vitro and then injected into mice caused the enhanced biosynthesis of TXA_2_ in vivo compared with the administration of HT29 cells not exposed to platelets [44] (Figure 3F). 

These findings suggest that platelets prime cancer cells to enhance their pro-thrombotic properties. Moreover, the injection into mice of cancer cells treated in vitro with platelets was associated with increased systemic biosynthesis of PGE_2._ This prostanoid elicits a wide range of cancer-related biological effects [45]. 

The enhanced systemic biosynthesis of TXA_2_ has been detected in colorectal cancer (CRC) patients *versus* controls, matched for sex, age, and cardiovascular risk factors [46]. The administration of a very low dose of aspirin (50 mg daily for five consecutive days) which mainly targets the platelet, caused a cumulative inhibition of platelet COX-1 activity either ex vivo, as assessed by the measurement of serum TXB_2_, or in vivo, as assessed by measuring urinary 11-dehydro-TXB_2_ (TX-M; a major urinary enzymatic metabolite of TXA_2_ in humans). Also, in patients with familial adenomatous polyposis (FAP being an inherited disorder characterized by the development of multiple noncancerous polyps in the colorectum at an early age that can progress to malignancy), the enhanced systemic biosynthesis of TXA_2_ was detected via a COX-1-dependent pathway since it was not affected by the selective COX-2 inhibitor celecoxib [47]. These data demonstrate that enhanced TXA_2_ biosynthesis occurs in vivo in intestinal neoplasia mainly from activated platelets and that treatment with the antiplatelet agent low-dose aspirin can prevent it. A controlled-release formulation of aspirin (such as 75 mg daily) [48], designed to inhibit maximally TXA_2_ production in platelets while sparing vascular prostacyclin (PGI_2_) biosynthesis, mainly derived from COX-2, was found to reduce the incidence and mortality of CRC [49]. This is consistent with the hypothesis that the antiplatelet effect of aspirin is central to its antitumor efficacy [6,7]. 

Dovizio et al. [43,47] have reported that HT29 cells trigger platelet TXA_2_ generation in vitro, which was almost completely inhibited by the pretreatment of platelets with aspirin. As previously reported, there is a multiplicity of molecular mechanisms that can be used by cancer cells to activate platelets and enhance TXA_2_ generation [50]. TXA_2_ is involved in the angiogenesis and development of tumor metastasis [51]. Thus, the pharmacological inhibition of the TXA_2_ synthase (TXAS) significantly inhibited tumor cell growth, invasion, metastasis, and angiogenesis in a range of experimental models [51]. 

In cocultures of human platelets and HT29 cells, Dovizio et al. [43] showed that the platelet-dependent induction of COX-2 in cancer cells, a hallmark of malignancy [52], was not affected by the selective inhibition of TXA_2_ generation in platelets by aspirin. HT29 cells were insensitive to the enhanced release of TXA_2_ by activated platelets due to undetectable levels of TP receptors in cancer cells [43]. These results provide the rationale for studying whether TP expression in circulating tumor cells can identify individuals who are responders to aspirin chemotherapy.

The enhanced biosynthesis of TXA_2_ in the murine colon-26 adenocarcinoma cell line (C26) via the introduction of the retroviral vectors carrying TXAS (TXA_2_ synthase) cDNA translated into faster growth and more abundant vasculature when inoculated to syngeneic BALB/c mice [53]. The administration of seratrodast, a TP antagonist, reduced the vasculature and tumor growth in C26-TXAS-derived tumors [53]. Matsui et al. [54] studied the role of TP signaling in the enhancement of tumor colony formation of B16F1 melanoma cells when intravenously injected into TP receptor knockout (KO) mice (TP KO) versus wild-type (WT) littermates. The TP KO mice showed a reduction in B16F1 lung colonization and mortality rate, which were associated with lower platelet counts. TP signaling regulated the P-selectin-mediated adhesion of activated platelets to P-selectin glycoprotein ligand-1 (PSGL-1) on metastatic tumor cells and endothelial cells and induced colony formation. Vascular endothelial growth factor (VEGF) and stromal-derived factor (SDF)-1 delivered from accumulated platelets induced the mobilization and recruitment of hemangiocytes expressing chemokine receptor type 4 (CXCR4) and vascular endothelial growth factor receptor 1 (VEGFR1) in the lung tissues [54]. Altogether these lines of evidence demonstrate the crucial role of TXA_2_ in cancer growth and the development of metastasis through several mechanisms involving the platelets and their interaction with the microenvironment and cancer cells. Low-dose aspirin can constrain cancer development and metastasis by affecting TXA_2_-dependent platelet activation [6,7]. However, the use of TP receptor antagonists could also affect the action of TXA_2_ when generated from cancer cells or in microenvironments. Appropriate clinical studies should test whether TP antagonism is more effective than low-dose aspirin as an anticancer agent. However, it should be considered that aspirin also inhibits platelet PGE_2_ generation, which plays numerous essential roles in cancer and metastasis development [44,45,55].

## 4. Role of EP Receptors in Cancer and the Effect of Antagonists

The mechanism involved in the capacity of platelets to induce a migratory/metastatic phenotype in cancer cells was explored by pretreating the platelets with aspirin in vitro [44]. Aspirin is an irreversible inhibitor of platelet COX-1 activity [22]. The drug prevented the capacity of platelets to induce EMT and enhance the migration of colon cancer cells via the inhibition of PGE_2_ released from platelets [44]. Using specific antagonists for the three EP receptors expressed in HT29 cells (EP1, EP2, and EP4, but not EP3), it was found that PGE_2_-dependent downregulation of E-cadherin in HT29 cells occurred through the activation of EP4 [44]. However, platelets expressed the EP3 receptor subtype, and DG-041, an antagonist of the EP3 receptor [56], prevented EMT and the migration in HT29 cells cocultured with platelets [44]. Altogether, these findings show the therapeutic potential of targeting EP4 receptors in cancer cells [57] and the EP3 receptor in platelets to prevent metastasis development. DG-041 was evaluated in phase IIa studies; however, its further development has recently been discontinued.

EP4 antagonists can restore antitumor immunity in the tumor microenvironment (TME), thus preventing tumor immune evasion caused by enhanced PGE_2_ [58]. Several companies are currently conducting clinical trials of EP4 receptor-selective antagonists for cancer therapy (ClinicalTrials ID: NCT03658772, NCT03696212, NCT03152370, NCT03661632) and evaluating EP4 antagonist therapy in anti-programmed death-1 (PD-1) refractory tumors, stable microsatellite tumors, and in combination with tumor radiotherapy. Recently, in a first-in-human study, E7046, a selective small-molecule antagonist of EP4, was orally administered to patients with selected advanced malignancies to define the appropriate dose to use in further clinical studies [59]. The results show that the molecule is manageable, tolerable, with immunomodulatory effects. A phase 1b study of E7046, at the doses of 250 and 500 mg, in combination with radiochemotherapy in patients with locally advanced rectal cancer is ongoing (ClinicalTrials ID: NCT03152370). 

Like EP4, EP2 receptors increase cAMP levels in the cell, which results in the activation of protein kinases (PKA) dependent transcription factors such as the cAMP-responsive element-binding protein (CREB). Moreover, the two receptors activate the GSK3β/β-catenin pathway, increasing the transcription of many genes implicated in cancer, such as c-myc, cyclin D1, and VEGF [60]. The majority of studies to date investigating the role of the EP2 receptor in malignancy have relied on gene deletion studies and gene knockout mice due to the lack of a selective antagonist [60]. However, a selective EP2 receptor antagonist, PF-04418948, has recently been developed, which may aid in the elucidation of the role of the EP2 [61]. 

The role of the EP2 receptor in cancer appears to be mostly ascribed to its induction of angiogenesis [62,63,64]. PGE_2_-induced EP2 receptor signaling also plays an important role in suppressing the antitumor immune response [65]. EP2 receptor activation by PGE_2_ markedly enhanced hepatocellular carcinoma cell invasion and migration ability by upregulating the expression level of Snail, a central inducer of EMT [66]. The EP2 receptor was linked to metastasis in breast cancer, partially through its ability to alter the response of cells to transforming growth factor-β (TGF-β) [67]. Recently, TPST-1495, an orally available dual antagonist that selectively blocks EP2 and EP4, was developed by Tempest Therapeutics. Preclinical data show that TPST-1495 is significantly more potent than a single EP4 antagonist against prostaglandin-mediated immune suppression and promotes antitumor efficacy. A first-in-human phase 1a/1b, multicenter, open-label, dose-escalation, dose, schedule optimization, and expansion study of TPST-1495 as a single agent and in combination with pembrolizumab is ongoing in order to determine its maximum tolerated dose (MTD) and/or recommended phase 2 dose (RP2D), safety, tolerability, pharmacokinetics, pharmacodynamics, and preliminary antitumor activity in subjects with advanced solid tumors (ClinicalTrials.gov Identifier: NCT04344795).

The role of the EP3 receptor in tumorigenesis is not clear. Conflicting effects on tumorigenesis following the targeting of the EP3 receptor have been reported [60]. The EP3 receptor exists as alternative-spliced variants, characterized by differences in the cytoplasmic C-terminal tail [68] and couples with several G-protein subunits, including G_i_, G_s_, and G_13_. The prominent EP3 splice variant couples to a G_i_ protein leading to the inhibition of AC and activation of the Ras/Raf and mitogen-activated protein kinase (MAPK) signaling pathway [69]. The expression of different EP3 isoforms and the conflicting results gained from suppressing EP3 receptor signaling indicates that the EP3 receptor is not a promising target for developing novel anticancer strategies. However, EP3 receptors expressed in the stromal cell compartment may play a role in tumor development by promoting angiogenesis and lymphangiogenesis [60]; thus, their blockage could indirectly affect tumorigenesis. Targeting EP3 in platelets with the antagonist DG-041 showed decreased thrombosis without impairing hemostatic competence [56], leading to the development of drugs with a better benefit-risk balance. This is a required expectancy for novel antiplatelet agents since enhanced bleeding is a side-effect of current antiplatelet agents. EP3 blockers could also prove helpful in interfering with the role of platelets in cancer metastasis. However, the clinical development of DG-041 appears to have been discontinued after phase II.

EP1 has a low affinity for PGE_2_ [70], and so it can be operative in the presence of the aberrant biosynthesis of COX-2-dependent PGE_2_ during tumorigenesis [52,55]. The EP1 receptor-selective antagonists ONO-8711 and ONO-8713 [71,72] conveyed the role of the receptor in intestinal tumorigenesis and other cancers, including breast [73] and skin cancer [74]. PGE_2_-EP1 receptor signaling is involved in cell migration and invasion and helps tumors to adapt to hypoxia [60]. In contrast, studies in breast cancer suggest that the EP1 receptor may have an antimetastatic function [75], with the nuclear expression of EP1 receptors correlating with good prognostic markers [76]. The reasons for these different findings are unclear. Given the multiple functions ascribed to PGE_2_-EP1 receptor signaling in cancer, the EP1 receptor may be a valid therapeutic target in some cancers. However, the data showing the in vivo efficacy of EP1 receptor antagonists were obtained via preclinical animal models, and no information is available on therapeutic benefits in human cancer.

In platelets, the effects of PGE_2_ are mainly the consequence of the interaction with EP3 and EP4 receptors, with EP3 receptors promoting platelet function and EP4 receptors inhibiting platelet function [77]. Thus, EP1 is not a target for developing antiplatelet agents able to affect the contribution of platelets to cancer metastasis.

## 5. Role of TP Receptors in Cancer and the Effect of Antagonists

The TP is a class A (rhodopsin class) G protein-coupled receptor identified in humans as a product of a single gene expressed in two splice variants, TPα and TPβ, containing an identical 328 amino acid sequence, but differing in their 15 amino acid and 79 amino acid sequences, respectively, in C-terminal regions, which may influence desensitization, internalization, and G protein coupling [78,79]. They have an identical ligand and similarly regulate phospholipase C, but they are differentially expressed throughout the body [78]. TPα (343 residues) is primarily expressed in platelets at high levels, while TPβ (407 residues) is mainly expressed in endothelial cells (ECs). In addition, the mRNAs for both splicing variants have been detected in vascular and lung smooth muscle cells (SMCs) and the brain. A functional role has been attributed to TP receptors in immune regulation [69]. While the privileged endogenous agonist is certainly TXA_2_, other natural/endogenous ligands, such as the prostaglandin-endoperoxide PGH_2_, other prostanoids, and isoprostanes (i.e., non-enzymatic AA metabolism products), may act through the interaction with TP receptors, though at higher concentrations [69,78]. Finally, TP receptors constitutively form homo- (TPa/TPa) and hetero-dimers between the two alternative mRNA splicing variants (TPa/TPb) or with the IP receptor, resulting in different ligand recognition and/or signal transduction [69].

TXA_2_ is a highly unstable product with a short half-life of about 30s. Therefore, it behaves as an autocrine or paracrine mediator in the tissues and cells near its production sites, and it is rapidly metabolized to the biologically inactive TXB_2_ [80]. TXA_2_ and its receptor have been widely implicated in a range of cardiovascular diseases due to their role in promoting platelet aggregation, vasoconstriction, and vascular proliferation [79]. TPa receptor expression and TXA_2_ production are increased in several cardiovascular and inflammatory diseases, while it is widely known that chronic inflammation contributes to the development of malignancies [81]. In common with other prostanoids, both TP and TXA_2_ synthase have been implicated in a variety of human cancer and metastasis such as lung [82], prostate [83], bladder [84], breast [85,86], and colon [87] (see also [88] for a review). Interestingly, a recent phenome-wide association study unveiled a TP receptor single nucleotide polymorphism (SNP, rs200445019) correlation with cancer metastasis across several cancer types. Accordingly, CPI211, a potent and selective antagonist of the TP receptor, potently blocked spontaneous metastasis from primary tumors without affecting tumor cell proliferation, motility, or tumor growth [89].

Reports on the involvement of the TXA_2_ system in tumor cell metastasis date back to 1982/3 [51,90]. Interestingly, while several tumor cells are known to stimulate platelet aggregation, causing the release of TXA_2_ (tumor cell-induced platelet aggregation, TCIPA) [91], the TP receptor-selective antagonist SQ29548 or the dual TXA_2_ synthase inhibitor/TP receptor antagonist BM-567 decreased platelet aggregation induced by osteosarcoma cells [92,93]. It has been demonstrated that TP receptor signaling facilitates tumor colonization through the P-selectin-mediated interaction of tumor cells with platelets and ECs [54], and, accordingly, aspirin which affects platelet COX-1-dependent TXA_2_ generation exerts an antimetastatic action by avoiding the enhanced pro-aggregatory effects induced by platelet-tumor cell interactions [44]. More recently, the genetic deletion or pharmacological inhibition of Pim kinases, known to be upregulated in several forms of cancer, reduced thrombus formation through the disruption of TXA_2_ receptor signaling [94].

In contrast, SQ29548 failed to inhibit both the AA-induced migration of breast cancer cells overexpressing COX-2 [95] and prostate tumor cell-induced platelet aggregation or secretion [96], in accordance with what has also been reported for aspirin in colorectal or breast cancer cells [97,98,99,100].

Even more controversial is the role of the TP receptor in angiogenesis, a fundamental process for cancer progression [101]. Several authors demonstrated that enhanced levels of TXA_2_ can induce endothelial cell migration and angiogenesis [102,103,104,105,106], and, accordingly, the inhibition of TXA_2_ biosynthesis reduced EC migration in vitro and angiogenesis in vivo [102,104]. The same effects were also obtained with the TP receptor antagonist SQ29548 [102]. In addition, IL-1β-induced EC migration and angiogenesis were also blocked by different TXA_2_ receptor antagonists (ONO-NT-126 and ONO-8809, in vitro and in vivo, respectively) [105].

Conversely, Asthon et al. and other groups found that TPb receptor stimulation reduces EC migration, intracellular communication, and in vitro capillary formation [107] as well as angiogenesis induced by VEGF [108,109] and fibroblast growth factor-2 (FGF-2) [110] in vitro and in vivo while also increasing apoptosis [111,112,113,114]. A possible explanation for these inconsistencies may lie in the animal model used, as only humans express the b isoform of the TP receptor [78], the overexpression of which in transgenic mice appears to reduce angiogenesis [108,110]. Indeed, as mentioned before, both isoforms share the same ligands (agonist and antagonists) and differ only in their C-terminal tails, making the mechanisms by which the two TP isoforms regulate angiogenesis still somewhat unclear [88]. In addition, the isoprostanes 8-iso-PGF_2α_, -PGE_2_, and -PGA_2_ have been shown to inhibit VEGF-induced migration and the differentiation of human coronary ECs [115], and, interestingly, their signaling is amplified by the presence of TPa-TPb heterodimers [116].

## 6. Role of Thrombin in Cancer and the Effect of Antagonists

In addition to its crucial role in the coagulation cascade, thrombin is involved in several aspects of cancer biology. It promotes the expression and activation of different integrins, including the α_IIb_β_3_ integrin (also known as GPIIb/IIIa) in many tumor cells [117]. Moreover, it simultaneously induces the release from platelets and the exposure on their surface of fibronectin and VWF, ligands of GPIIb/IIIa, thus promoting the bridge-building between platelets and tumor cells [118].

Thrombin actions on target cells occur through the interaction with PARs by cleaving their N-terminal end, thus inducing cell activation. PARs are expressed both by platelets and tumor cells. The high-affinity PAR-1 and the low-affinity PAR-4 are expressed on human platelets [31]. The thrombin-mediated PAR-1 and PAR-4 activation of platelets induces aggregation and the release of pro-angiogenic and mitogenic mediators (such as PDGF, VEGF, and angiopoietin-1). It has been shown that the PAR-1 stimulation of platelets promotes the migration of endothelial progenitor cells and the formation of new vessels, which are important events for metastasis formation [119]. In vitro studies have shown that thrombin-mediated PAR-1 activation increases the migration of A-498 human renal carcinoma cells through the activation of protein kinase C (PKC), MAP kinases, transcription factor NF-kB, and cAMP-dependent protein kinases (PKAs) [120].

Moreover, osteosarcoma cells (U2-OS) showed an increased capacity to invade a Matrigel barrier in vitro after stimulation with thrombin or the PAR-1 thrombin receptor-activating peptide (TRAP) [121]. The exposure of different cancer cell lines to platelets pre-treated with thrombin enhances tumor cell adhesion [122]. Finally, in mice, thrombin injected intravenously together with different syngeneic tumor cell lines significantly increased pulmonary metastasis formation [122,123]. The use of siRNA (short interfering RNA) encapsulated liposomes to inhibit PAR-1 in tumor cells was associated with a reduced expression of angiogenic and metastatic genes (such as VEGF, Interleukin (IL)-8, and matrix metalloproteinase-2 (MMP-2)) and blood vessel density. These results support the role of PAR-1 in the regulation of melanoma cell growth and metastasis by affecting both invasive and angiogenic factors. 

Altogether, results from in vitro and in vivo studies support the development of anticancer agents which directly or indirectly inhibit thrombin or its receptors (PARs). Selective PAR-1 antagonists (SCH79797, vorapaxar, atopaxar) can block the receptor activation on tumor cells, platelets, fibroblasts, and the endothelium. Direct thrombin inhibitors, such as dabigatran, act as anticoagulants (delaying blood clotting) by directly inhibiting the enzyme thrombin. Pepducin technology was developed to target receptor G-protein interactions at the plasma membrane interface. Palmitoylation or other lipid moieties attached to the peptide partitions the lipopeptide across the plasma the bilayer. The use of cell-penetrating pepducin inhibitors, generated against the first (i1) and third (i3) intracellular loops of PAR-1, significantly inhibited cell migration in primary and established lung cancer cell lines, similarly to the silencing of PAR-1 expression with short hairpin RNA [124].

## 7. Adenine Nucleotides and Purinergic Receptors

Extracellular (e) adenine nucleotides, both extracellular eADP and eATP, are deeply involved in the modulation of hemostasis. Platelets have a small number of dense granules, usually three to eight. Unlike alpha granules, which hold a cargo with hundreds of proteins, these contain calcium, serotonin, and a non-metabolic pool of ADP and ATP [125]. Once released, following the action of primary platelet agonists such as thrombin and collagen, these adenine nucleotides, and their nucleoside derivative adenosine (Ado), act as recognized modulators of several platelet functions, including aggregation, shape change, and the release of alpha granules [126,127,128]. Extracellular ADP is a weak agonist of platelet aggregation compared to thrombin and collagen in that it triggers only reversible platelet responses. However, this nucleotide is recognized as a key “secondary” agonist able to cause a significant amplification of platelet responses and to contribute to thrombus stabilization [129]. 

Under pathological conditions including hypoxia, ischemia, inflammation, and cancer, eATP concentrations have been reported to rise significantly [130,131]. They are modulated by the activity of ectoenzymes called ectonucleotidases, which metabolize nucleotides into the respective nucleosides [132]. Thanks to the use of the pmeLUC probe, a plasma membrane-expressed luciferase tool targeted to the outer side of the plasma membrane [133,134], it has been possible to reliably measure the eATP levels, which in the tumor microenvironment of several cancer types reaches millimolar concentration [134,135]. Extracellular adenine nucleotides and nucleosides exert their biological effects by interacting with specific plasma membrane receptors. These sites are divided into two major classes named P1 and P2 purinergic receptors based on their preferential binding by Ado (P1) or adenine and uracil nucleotides (P2) [136]. The platelets express four major P2 receptor subtypes, the P2Y1, P2Y12, and P2Y14 metabotropic receptors, and the ligand-gated ion channel P2X1 sites [137]. Although P2Y1-selective antagonists have been identified, at present, the only Food and Drug Administration (FDA) and European Medicines Agency (EMA) approved platelet purinergic receptor antagonists are a group of drugs targeting the P2Y12 sites, which includes: (i) thienopyridines such as ticlopidine, clopidogrel, and prasugrel, which irreversibly inhibit P2Y12 upon metabolic conversion into active metabolites via the hepatic cytochrome P-450 system [138,139]; and (ii) ticagrelor, cangrelor, and elinogrel, which reversibly and directly bind the receptor [138,139].

P2Y12 shares with the P2Y1 receptor the canonical seven-transmembrane helical architecture of GPCRs. The human purinergic receptor is encoded by a gene located on chromosome 3q25.1 and contains 342 amino acid residues [140,141]. The structure of P2Y12 shows two N-linked glycosylation sites at its extracellular amino terminus, which can serve as modulators of its activity (for a comprehensive review of the structure of P2Y12 see [21]). P2Y12 receptor expression was firstly reported to be restricted to platelets, about 400 sites/platelet [142], where it represents a key regulator of eADP-mediated physiological platelet aggregation [21]. Besides platelets, this purinergic receptor was subsequently shown to play a role also in the control of other cell functions, including: (i) microglia, where P2Y12 mediates the nucleotide-induced microglial chemotaxis [143] and the NLRP3 inflammasome to enhance microglial inflammation [144]; (ii) osteoclasts, where it mediates eADP-increased cell adhesion and resorptive activity [145]; (iii) leukocytes, eosinophils, macrophages, and dendritic cells, where P2Y12 mediates inflammatory and immune response [146,147,148,149]; and (iv) vascular smooth muscle cells (VSMCs), where its activation causes vasoconstriction [150] and induces VSMC motility and migration [151]. The P2Y12 receptor couples to the Gαi2 protein subtype, and its activation leads to the inhibition of cyclic (c) AMP production [152]. This, in turn, causes a reduction in the cAMP-dependent PKA–mediated phosphorylation of downstream effectors such as vasodilator-stimulated phosphoprotein (VASP) [152], which in platelets restrains either secretory or adhesive events. A flow cytometry-based assay which measures the extent of VASP phosphorylation is used to monitor platelet responsiveness to P2Y12 targeted antiplatelet therapy (particularly in tailoring the treatment with the oral P2Y12 inhibitor clopidogrel) [129,153,154]. Extracellular ADP acts as the endogenous native agonist at the P2Y12 receptor, whereas eATP and its analogs behave as partial agonists or antagonists at this receptor subtype [155]. In platelets, the eADP-P2Y12- Gαi2 signaling pathway contrasts the antiplatelet effect mediated by PGI_2_ and eAdo, activating the PGI_2_ receptor IP and the adenosine receptors A2A and A2B, respectively, inhibiting platelet function. This mechanism contributes to the overall antithrombotic effects of P2Y12 receptor antagonists and underlies the more severe bleeding observed in P2Y12R-KO mice compared to P2Y1R-KO animals [21]. In 2008, Guidetti and co-workers pointed out that AR-C69931MX, a P2Y12 specific receptor antagonist inhibited eADP-induced phosphorylation of pleckstrin [156]. This is a modular platelet protein involved in granule secretion, actin polymerization, and aggregation and, in platelets, is the primary PKC substrate [157]. Several lines of evidence indicate the crucial role of phosphoinositide 3-kinase (PI3K) in the signaling pathway triggered by the P2Y12 receptor. Its activation has been shown to recruit G_bg_subunits causing PI3K dependent Akt phosphorylation and Rap1b activation, a critical positive regulator pathway for the integrin GPIIb/IIIa. In this way, the sustained activation of P2Y12 contributes to thrombus stabilization. Among the different PI3K isoforms, the β one appears to be crucial for ADP-induced TXA2 generation and platelet aggregation [158] and cooperates with PI3Kγ isoform in sustaining integrin activation [159,160]. P2Y12 is the target of numerous effective antithrombotic agents divided into two groups, the irreversible antagonists ticlopidine, prasugrel, and clopidogrel, which have been included in the World Health Organization’s List of Essential Medicines 2021, and reversible ones, which include ticagrelor, cangrelor, and elinogrel. 

## 8. Role of the P2Y12 Receptor in Cancer and the Effect of Antagonists

A role of the P2Y12 ADP receptor in sustaining and promoting the metastatic process first emerged from results obtained by treating rodents injected with a B16 melanoma or AH130 rat ascites hepatoma cells [161] with ticlopidine. The antiplatelet drug (100 mg/Kg) administered per os using a stomach tube 3 hr before tumor cell inoculation suppressed the formation of pulmonary nodules. Ticlopidine was also able to inhibit the spontaneous pulmonary metastasis of Lewis lung carcinoma [161]. These data were confirmed using a mouse model of spontaneous induced lung metastasis, obtained by injecting Lewis lung carcinoma (LLC), where the absence of P2Y12 significantly reduced pulmonary metastasis [162]. P2Y12 deficiency diminished the capability of cancer cells to stimulate the production of active TGFb1 from platelets, with this resulting in the prevention of the platelet-induced EMT of LCC cells [162]. 

Similarly, in HT29 human colon carcinoma cells, in vitro exposure to platelets leads to the induction of EMT in tumor cells [44]. This was associated with enhanced cell mobility and a higher incidence of lung metastasis when the pre-exposed HT29 were inoculated into the tail vein of humanized immunodeficient mice when compared to untreated HT29 cells [44]. These effects were inhibited when the cells were co-cultured with platelets in the presence of ticagrelor or aspirin [44]. The P2Y12 antagonist also constrained TXB_2_ and PGE_2_ production, indicating an inhibitory effect on the release of arachidonic acid from platelet membrane phospholipids [44]. 

In the presence of eADP, ticagrelor also significantly reduced the ability of platelets to bind to three different breast cancer cell lines, namely MCF-7, MDA-MB-468, and MDA-MB-231 human mammary carcinoma cells [163]. Moreover, ticagrelor (10 mg/kg), but not clopidogrel (10 mg/kg) or saline, administered intraperitoneally in an orthotopic metastasis model obtained by the subcutaneous inoculation of 4T1 mammary carcinoma cells, caused a reduction in metastasis formation and an improvement in survival. The authors hypothesize that clopidogrel failure in this setting could be due to its lower safety profile and to myelotoxicity caused by its metabolic products, in particular [164]. The efficacy of ticagrelor was reported in different metastasis models irrespective of cancer origin. The P2Y12 antagonists reduced lung and liver metastasis by about 85% in mice that had received intrasplenic or tail vein injections of B16-F10 melanoma cells [164]. Also, in these settings, the drug improved the rate of animal survival compared to controls.

Similarly, in a rodent 4T1 breast cancer model, ticagrelor reduced metastasis formation in lung and bone marrow by 55% and 87%, respectively [164]. Ticagrelor was also reported to reduce the growth of primary tumors in murine models of ovarian cancer generated by the intraperitoneal injection of cancer cells [165]. In this setting, the P2Y12 antagonist, given by gavage at a dosage of 100mg/Kg, restrained tumor growth by 60% compared with aspirin administered at 150 mg/kg and by 75% compared with a vehicle (phosphate-buffered saline, 200 mL). The knockdown of CD39 on ovarian cancer cells caused an increase in tumor growth in cancer-bearing mice. Ticagrelor, as well as the recombinant CD39, was able to reduce the increased cancer cell proliferation induced by platelets. Thus, both P2Y12 receptor and ADP levels in the milieu surrounding cancer cells and platelets were involved in the growth of primary tumors in this in vivo cancer model [165].

A more direct role of these purinergic receptor subtypes in cancer growth and metastasis cannot be ruled out beyond an indirect effect mediated by platelet P2Y12. Although the expression of P2Y12 in cancer cells has been poorly investigated, the receptor protein has been found in several kinds of cancer cells, including glioma and astrocytoma cells, different human pancreatic cancer cell lines, and human melanoma tumor-associated macrophages. It can trigger biological events such as MAPK activation, EGFR and Akt phosphorylation, cell proliferation, and autophagy [166,167,168]. 

P2Y12 receptor antagonists, combined with aspirin (dual antiplatelet therapy, DAPT), still represent the cornerstone of antithrombotic therapy for secondary prevention in patients with acute coronary syndromes or who receive percutaneous coronary intervention (PCI) [169,170,171]. Although concerns had been raised on the possible association between P2Y12 antiplatelet therapy and solid tumor growth or metastatic dissemination, either follow-up clinical trials or meta-analyses did not point out a significant association between the use of P2Y12 receptor antagonists and cancer incidence or cancer-related death [172,173,174,175]. Moreover, the investigation carried out by Leader and co-workers on a cohort including 183,912 individuals treated with aspirin, either alone or in association with clopidogrel and nonusers, showed that the risk of cancer was lower in subjects exposed to DAPT (HR 0.92; 95% CI, 0.86-0.97) [175]. These results suggest that clopidogrel may reduce cancer incidence. This hypothesis was strengthened by a nested case-control study on 15,491 incident cases of CRC and 60,000 controls from a primary care database in Spain (Base de datos parala Investigación Farmacoepidemiológica en Atención Primaria) [176]. The study reported that clopidogrel (75 mg daily) reduced the risk of CRC. This effect was particularly evident after the first year of drug treatment (OR, 0.65; 95%CI, 0.55–0.78), supporting the hypothesis that the inhibition of P2Y12 may represent an effective anticancer therapeutic approach [176]. Recently, the results of the TICONC (Ticagrelor-Oncology) study have been published; this is the first study assessing whether ticagrelor may also inhibit platelet activation in cancer and constrain metastasis formation. The study was divided into two phases, one in vitro and the second in a clinical trial. In the in vitro study, the pre-treatment of platelets with ticagrelor (10 mM) significantly reduced large platelet–platelet aggregates induced by MCF-7 and HT29 cancer cells, with more platelets remaining dispersed throughout the sample. The pre-incubation of platelets with ticagrelor almost halved the platelet population expressing annexin V or phosphatidylserine increased by platelet exposure to both cancer cell lines. Platelets were also assessed for their capability to induce cancer cell arrest and adhesion to the endothelial wall, a recognized crucial mechanism underlying metastatic disease progression. The addition of platelets to HT29, but not to MCF-7 cancer cells, significantly increased cell adhesion to the cultured human umbilical endothelial vein. The pre-treatment of platelets for 10 min with ticagrelor (10 mM) reduced HT29 cell adhesion from 25.2 ± 4.6% to 17.9 ± 4.5% (*p* = 0.020; n = 5). A similar reduction was obtained using aspirin (50 mM), and the pre-treatment of platelets with both antiplatelet agents was not more effective than drugs given alone [177]. The study’s second phase consisted of a randomized crossover clinical trial involving 38 subjects: 22 healthy controls, 10 patients with metastatic breast cancer, and 10 with metastatic colorectal cancer. After screening, each population was randomized and received low dose aspirin (75 mg daily) or ticagrelor (90 mg twice daily) for two weeks, followed by a two-week washout period. This was followed by another two weeks of monotherapy (crossover) and, finally, two weeks of DAPT. Blood samples were collected and assayed for platelet activation and inhibition by aggregometry and flow cytometry at each visit. The results fit with those of the in vitro phase. Cancer patients treated with ticagrelor showed significantly reduced spontaneous platelet aggregation and activation levels compared with the baseline. The study’s limitations are mainly the sample size and the evaluation of only two types of cancer. However, the finding that ticagrelor alone, not in dual therapy, was more effective than aspirin in counteracting both in vitro and in vivo the cancer cell-mediated platelet aggregation is a proof-of-concept, and further investigations based on larger randomized studies are needed for acquiring unequivocal answers on the possibility of using ticagrelor monotherapy to: (i) prevent/counteract cancer-associated thromboembolism, (ii) reduce metastasis formation, and (iii) improve progression-free survival. Recently, the adenosine tetraphosphate derivative GLS-409, which synergistically inhibits both human platelet P2Y1 and P2Y12 receptors and effectively antagonizes ADP-mediated human platelet aggregation, has been proposed as a novel potential antiplatelet drug [168,169]. In a canine model of recurrent coronary thrombosis, this compound, at the lower dose used (0.00054 mg/kg bolus + 0.000018 mg/kg/min infusion for 2 h), inhibited platelet-mediated thrombosis without causing an increase in bleeding time measured immediately before and after the 2-h treatment period [178]. This characteristic should make GLS-409 particularly attractive in terms of its role as a novel therapeutic approach targeting platelet-mediated chronic inflammation such as cancer. The novel dual drug also showed a reversible mechanism in inhibiting human platelet functions, as shown by the rapid recovery of platelet reactivity to ADP [179]. It should be of interest to assess, in both in vitro and in vivo studies, the capability of this drug to constrain platelet-mediated tumorigenesis and metastasis.

## 9. Development of Novel Antiplatelet Agents Targeting Intracellular Signaling Pathways

### 9.1. PI3Kβ Inhibitors

In platelets, PI3Kβ appears to play a major role in regulating signals downstream of the G_i_-coupled P2Y12 receptor necessary for the activation of Rap1b (ras-related protein1, a molecule switching from an inactive GDP-bound state and an active GTP-bound state) [160]. This pathway is involved in sustained integrin α_IIb_β_3_ activation. PI3Kβ inhibitors have been shown to affect in vitro platelet aggregation and in vivo thrombus generation without causing a significant increase in bleeding [180,181]. 

AZD6482 is a PI3Kβ inhibitor in clinical development which has been shown to affect platelet aggregation in vitro in response to ADP, TRAP, and collagen; 3-h parenteral infusion of AZD6482 in humans was well tolerated, and there was no change in bleeding time [182,183]. However, the potential influence on insulin signaling should be considered. AZD6482 might be an effective antithrombotic agent in managing patients undergoing cardiopulmonary bypass surgery and stroke, also in combination with aspirin [184]. 

### 9.2. G_q_ Inhibitors

YM-254890, a cyclic depsipeptide discovered in Chromobacterium sp. QS3666 culture broth, is a potent inhibitor of ADP-induced platelet aggregation, acting as an inhibitor of G_q/11_ [185]. The compound also attenuated collagen-, TRAP-, AA-, and U46619-induced platelet aggregation. YM-254890 showed antithrombotic effects in a model of cyclic flow reductions in the femoral artery of cynomolgus monkeys without affecting systemic blood pressure or prolonging bleeding time. Recently, Peng et al. [186] found that YM-254890 is not a selective inhibitor for the G_q_ protein and that instead it acts as a broader spectrum inhibitor for G_q_, G_s,_ and G_i_ with high potency, without affecting non-GPCR-mediated cellular signaling. 

Despite G_q_ being an attractive pharmacological target, detailed studies should be performed to verify the possible toxicity of the inhibitors since this signaling pathway has a widespread influence on many GPCRs expressed in multiple organs.

## 10. Conclusions

The prevention of cardiovascular disease is obtained via drugs affecting the amplification pathways of platelet aggregation, i.e., aspirin at low doses, which mainly targets the platelet COX-1 and inhibits TXA_2_, and P2Y12 antagonists, which block the action of ADP [154]. However, these drugs are associated with an enhanced risk of bleeding, and the development of safer drugs is an unmet medical need. Moreover, an inter-subject variability in the response to the existing drugs has been described [187]. 

The rationale behind the development of specific TP antagonists was to avoid the marginal reduction in vascular PGI_2_ biosynthesis observed on 100 mg/day aspirin [188] and the aim of blocking TP activation not only by TXA_2_ but also by unconventional ligands, such as the isoprostane products of lipid peroxidation [189]. However, platelet activation is not always associated with concurrent changes in isoprostane biosynthesis [190], and the contribution of sparing 10–20% of vascular PGI_2_ biosynthesis appears not to be clinically relevant. The finding of similar GI bleeding rates associated with low-dose aspirin and the TP antagonist terutroban [191] suggests the involvement of the inhibition of TXA_2_-dependent platelet function in these side effects. Moreover, PGH_2_ (the product of COX-1) can substitute for TXA_2_ as an agonist of the TP [192], limiting the clinical efficacy of TXA_2_ synthase inhibitors. Thus, the development of TXA_2_ synthase inhibitors was abandoned after several inhibitors reached human trials [192]. 

However, several pieces of evidence suggest that platelet-derived TXA_2_ exerts additional functions beyond thrombosis. Thus, Sacco et al. [193], using a mouse with a specific deletion of COX-1 in platelets/megakaryocytes, showed that platelet-derived TXA_2_ may play an essential role in the development of intestinal chronic inflammation and fibrosis through the induction of fibroblasts with proliferative, migratory, and mesenchymal features, which may promote a persistent proinflammatory state in the colonic mucosa. Moreover, Guillem-Llobat et al. [44] showed the role of platelet-derived PGE_2_ in inducing a migratory phenotype associated with EMT in cancer cells. Aspirin can prevent these effects by blocking both prostanoid pathways, while selective blockers of TP or EPs did not and should cause fewer antitumor effects. 

The role of COX-2-dependent PGE_2_ in cancer is sustained by a large amount of experimental evidence [194] and by the results of clinical studies with coxibs [6]. However, the chronic use of coxibs is not recommended because of their significant cardiovascular toxicity [34]. EP receptor antagonists, particularly those targeting the EP1, EP2, and EP4 receptors, have been used successfully in preclinical models to suppress the development and growth of tumors and metastasis [60]. However, their anticancer effects and safety profile have not been evaluated in randomized clinical trials. One limitation is that EP receptor antagonists target only one pathway; thus, more than one antagonist may be required to suppress and/or treat malignant disease and have a comparable efficacy to coxibs. 

Several lines of preclinical data support the notion that P2Y12 receptor antagonists, including clopidogrel, ticagrelor, and prasugrel, might represent potential anticancer agents, in addition to their role as effective antithrombotic drugs. However, further studies are required in experimental animals and patients before any recommendation for using P2Y12 antagonists in cancer prevention and progression can be made. In a nested case-control study of a primary care database in Spain, Rodríguez-Miguel et al. [176] found that clopidogrel use, alone or in combination with low-dose aspirin, reduced CRC risk by 20% to 30%, a magnitude similar to that of low-dose aspirin alone. These data support the concept that inhibiting platelets is an effective strategy for preventing CRC. These findings provide the rationale for performing large randomized clinical trials with P2Y12 antagonists alone and combined with low-dose aspirin to achieve robust clinical evidence of their efficacy before recommending their use in cancer patients.

## Data Availability

Not applicable.
